# Effectiveness of protocol-based pharmacotherapy management collaboration between hospital and community pharmacists to address capecitabine-related hand–foot syndrome in cancer patients: a retrospective study

**DOI:** 10.1186/s40780-021-00191-1

**Published:** 2021-03-01

**Authors:** Nobuhiko Nakamura, Hiroki Shiraiwa, Yasuhiro Haruna, Tomoki Ichijima, Tomoko Takeda, Koji Hasegawa, Masaaki Kusumoto, Yoshitaka Yano

**Affiliations:** 1 Education and Research Center for Clinical Pharmacy, Kyoto Pharmaceutical University, 5-Nakauchi-cho, Misasagi, Yamashina-ku, Kyoto, 607-8414 Japan; 2 Department of Pharmacy, Kyoto Chubu Medical Center, 25-Yagiueno, Yagi-cho, Nantan City, Kyoto, 629-0197 Japan

**Keywords:** PBPM, Community pharmacists, Hospital pharmacists, Pharmaceutical care, Hand–foot syndrome, Capecitabine, Japan

## Abstract

**Background:**

Pharmaceutical care of capecitabine-related hand–foot syndrome (HFS) is extremely important to avoid the progression of the syndrome. Protocol-based pharmacotherapy management (PBPM) of HFS by community pharmacists has been introduced in our community, whereby the community pharmacist instructs patients to use steroid creams if they develop HFS of grade 2 or higher. This study aimed to evaluate the effectiveness of PBPM in cancer patients with HFS by comparing it to conventional pharmaceutical care using monitoring reports for pharmacotherapy management by community pharmacists.

**Methods:**

From September 2017 to August 2019, we retrospectively investigated the medical records of 396 cancer patients who received capecitabine adjuvant chemotherapy. Before PBPM implementation, conventional pharmaceutical care was administered from September 2017 to August 2018; these patients served as the control group. Care was switched to PBPM in September 2018, and PBPM was applied from September 2018 to August 2019; these patients served as the PBPM group. We excluded patients who received both conventional pharmaceutical care and PBPM. We categorized all cases into two groups: age ≤ 69 years and age ≥ 70 years.

**Results:**

In all, 396 cases were included, of which 227 were ineligible, such as those of cancer patients who received both conventional pharmaceutical care and PBPM. Among patients aged higher than 70 years, the incidence and severity of HFS associated with PBPM were significantly lower than those associated with conventional care (grade 0: 59.5% [44/74] vs. 30.6% [11/36], grade 1: 33.8% [25/74] vs. 63.9% [23/36]). All patients continued to receive the capecitabine, HFS severity improved to grade 1 during the study period, and treatment of HFS was not stopped.

**Conclusion:**

Our findings suggest that PBPM is effective for addressing capecitabine-related HFS among cancer patients aged higher than 70 years, in that it helps prevent an increase in HFS severity.

## Background

Capecitabine is an extensively used oral anticancer drug for patients with metastatic breast cancer, adjuvant colorectal cancer, and gastric cancer in Japan [1–3]. Oral capecitabine plus oxaliplatin (XELOX) has been shown to be non-inferior to FOLFOX-4, which comprises leucovorin calcium, fluorouracil, and oxaliplatin, as first-line therapy for metastatic colorectal cancer [4, 5]. Furthermore, XELOX requires fewer planned clinic visits than does FOLFOX, because oxaliplatin is administered every 3 weeks (rather than every 2 weeks) and capecitabine is received orally [5]. However, capecitabine has been frequently reported to be associated with hand–foot syndrome (HFS). HFS is characterized by redness, marked discomfort, swelling, and tingling in the palms of hands and/or the soles of feet. Symptoms may vary from relatively painless to severely painful [6]. The mechanism for capecitabine-induced HFS appears to be related to the accumulation of 5-FU metabolites in the skin, much remains to be determined [7]. In fact, it was reported that HFS of any grade is observed in 30 to 40% of patients receiving XELOX with/without bevacizumab for metastatic colorectal cancer [4, 5]. However, treatment efficacy was not compromised in patients in whom dose reduction was required owing to adverse events [8, 9].

In Japan, the means of the age diagnosed with colon and rectal cancers were 67.4 and 65.5 years old, respectively [10]. For elderly patients aged > 75 years with metastatic colorectal cancer, it was reported that XELOX with bevacizumab is safe and effective in terms of PFS and OS [11]. Analysis of the adherence issues in relation to the patients’ age showed a trend toward worse adherence to capecitabine therapy in the group of patients aged ≥80 years [12]. However, the severe of HFS with capecitabine has not enough examined in elderly patients. Therefore, in patients undergoing cancer chemotherapy with XELOX, symptomatic treatment based on periodic monitoring of skin symptoms by community pharmacists may be required to minimize the risk of severe HFS.

An oral chemotherapy management service provided by pharmacists has been reported to be effective in delivering early interventions, resulting in decreased rates of adverse effects, nonadherence, drug interactions, and medication errors over time [13]. In a previous pharmacist-led study, telephonic follow-up was found to have slightly improved patients’ treatment adherence and overall survival [14]. However, no study has assessed the effectiveness of pharmacotherapeutic management of adverse events in cancer patients receiving capecitabine by community pharmacists. Hence, it remains unclear whether protocol-based pharmacist-led care is more effective than conventional care in managing these adverse effects in this patient population.

In Kyoto Chubu Medical Center, protocol-based pharmacotherapy management (PBPM) has been established for capecitabine-related HFS in cancer patients. PBPM requires pharmacists to determine the severity of skin symptoms based on Common Terminology Criteria for Adverse Events (CTCAE) grading and provide early and appropriate usage instructions for topical steroid drugs. However, it was noted that supportive care for adverse events was not administered promptly because of delays in patients’ hospital visits when community pharmacists assessed skin condition in cases in which HFS severity was of grade 2 or higher. Hence, it is necessary to consult a physician newly for cancer patients to be treated with a topical steroid drug. In light of this background, this study was aimed at evaluating the effectiveness of PBPM compared to conventional pharmaceutical care in addressing capecitabine-related HFS among cancer patients.

## Methods

### Patients and setting

From September 1, 2017, to August 31, 2019, we retrospectively investigated 396 cancer patients who received capecitabine adjuvant chemotherapy at Kyoto Chubu Medical Center, Kyoto, Japan. Before PBPM implementation, conventional pharmaceutical care was applied for 1 year from September 2017 to August 2018. We switched to PBPM in September 2018, and PBPM was applied for 1 year from September 2018 to August 2019. We excluded cancer patients who received both conventional pharmaceutical care and PBPM.

### Prevention and treatment using PBPM

**Fig. 1:**
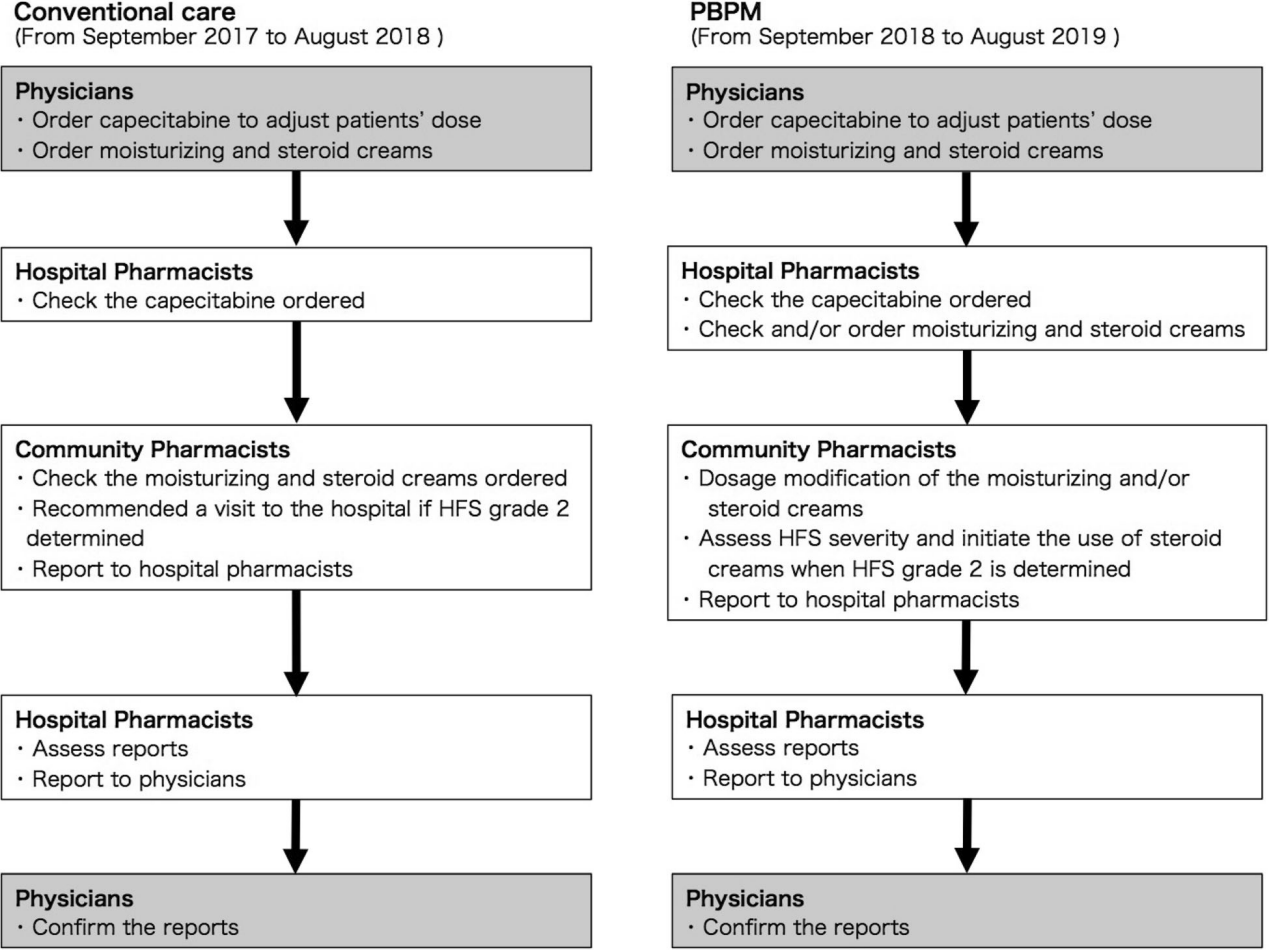
Conventional care and protocol-based pharmacotherapy management (PBPM) workflows of pharmacists

Pharmacists, physicians, and university faculty were involved in the development of PBPM for adverse event prevention and treatment with reference to current guidelines and literature. After the physician ordered the capecitabine chemotherapy, the hospital pharmacist ordered moisturizing and steroid creams substituting for physicians based on the PBPM. The dosages of the moisturizing and steroid creams were modified according to patients’ preferences and the physician’s approval was sought later. Community pharmacists managed the capecitabine-related HFS based on the PBPM approximately once a week by phone or face-to-face at the pharmacy counter. The severity of HFS was classified according to the CTCAE v.4.0. In addition, the community pharmacist recommended the appropriate protocol to visit a hospital when the patient developed severe HFS. The community pharmacist reported the HFS severity assessments to the hospital pharmacist in Kyoto Chubu Medical Center. The hospital pharmacist recorded the severity assessments in the electronic medical record systems, which was ratified by the physician later. The physician retained all responsibility for patient management (Fig. 1).

### Outcome measurements

The primary outcome was to evaluate the efficacy of PBPM to address capecitabine-related HFS in cancer patients by comparing it to conventional pharmaceutical care using monitoring reports for pharmacotherapy management by community pharmacists. Basic information regarding patients’ characteristics, including gender, age, Eastern Cooperative Oncology Group (ECOG) performance status score, primary site of cancer, and laboratory data, excluding neoadjuvant or adjuvant, palliative were collected from medical records. We evaluated the grade of HFS, constipation, diarrhea, nausea/vomiting, stomatitis, loss of appetite, fatigue, rash, fever, watering eyes, hypertension and peripheral neuropathy. We also evaluated the grade of HFS each regimen between PBPM group and control group. We performed receiver operating characteristic (ROC) curve analysis in order to determine the cutoff value of the age.

### Statistical analysis

The difference in the severity of capecitabine-related HFS before and after the implementation of pharmaceutical care based on the PBPM was compared using the Wilcoxon signed-rank test. Data were analysed by using the Statistical Package for the Social Sciences for Windows (SPSS-11 version II; SPSS, Chicago, IL, USA). The results with a *p*-value of < 0.05 were considered significant.

## Results

In all, 396 cases were included, of which 227 were ineligible, such as those of cancer patients who received both conventional pharmaceutical care and PBPM. After exclusion of these 227 ineligible cases, we finally analysed 169 cases (59 and 110 cases in the control and PBPM groups, respectively). Table 1 shows the characteristics of the control and PBPM groups. The median age of both groups was 71.0 years (control group range: 60.0–84.0 years, PBPM group range: 49.0–89.0 years). In most cases in both groups, the adjuvant chemotherapy regimen was capecitabine combined with oxaliplatin (XELOX) rather than capecitabine alone.

**Table 1:** Characteristics of each group

Characteristics	Control group (*n* = 59)	PBPM group (*n* = 110)	*p*-value
Gender			0.5033
Male, n (%)	40 (67.3)	80 (72.7)	
Female, n (%)	19 (32.2)	30 (27.3)	
Age (year), median (range)	71.0 (60.0–84.0)	71.0 (49.0–89.0)	0.6601
ECOG performance status, n (%)			2.8390
0	37 (62.7)	90 (81.8)	
1	17 (28.8)	9 (8.2)	
2	5 (8.5)	1 (0.9)	
Primary site of cancer, n (%)			0.0007
Colorectal	53 (89.8)	110 (100)	
Breast	5 (8.5)	–	
Stomach	1 (1.7)	–	
BSA (m^2^), median (range)	1.70 (1.31–1.97)	1.62 (1.24–2.05)	0.0301
Daily dose of capecitabine, median (range)	3000 (1200-3600)	2400 (1200-4200)	0.5894
Chemotherapy regimens, n (%)			0.2509
XELOX	34 (57.6)	55 (50.0)	
XELOX + Bevacizumab	16 (27.1)	30 (27.3)	
Capecitabine	9 (15.3)	24 (21.8)	
XELIRI + Bevacizumab	0 (0)	1 (0.9)	
Laboratory data, median (range)			
Cr (mg/dL)	0.69 (0.45–1.6)	0.75 (0.37–1.51)	0.1497
GFR (mL/min/1.78m^2^)	76.8 (32.5–105.8)	72.4 (6.5–143)	0.2323
AST (U/L)	31 (16–84)	23 (11–71)	2.4730
ALT (U/L)	21 (9–47)	15 (6–74)	0.0046
WBC (/μL)	4490 (2750-8070)	4145 (2610-10,540)	0.0097
Hb (g/dL)	12.1 (6.3–16.6)	12.0 (9.0–18.3)	0.6454
RBC (10^6^/μL)	3.81 (1.7–5.4)	3.9 (2.2–5.9)	0.1067

Abbreviation: ECOG, Eastern Cooperative Oncology Group: BSA, body surface area: XELOX, capecitabine plus oxaliplatin: XELIRI, capecitabine plus irinotecan: Cr, creatinine: GFR, glemerular filtration rate: AST, aspartate aminotransferase: ALT, alanine aminotransferase: WBC, white blood cell: Hb, hemoglobin: RBC, red blood cell

**Table 2:** Adverse events with capecitabine adjuvant chemotherapy

Adverse events	Control group (*n* = 59)					PBPM group (*n* = 110)					*p*-value
Chemotherapy regimens	All (%)	Grade 0	Grade 1	Grade 2	Grade 3	All (%)	Grade 0	Grade 1	Grade 2	Grade 3	
Hand-foot syndrome	59 (100)	25	31	3	0	110 (100)	66	39	5	0	0.0382
XELOX	34 (57.6)	16	17	1	0	55 (50.0)	33	20	2	0	0.2720
XELOX + Bevacizumab	16 (27.1)	6	9	1	0	30 (27.3)	18	12	0	0	0.1162
Capecitabine	9 (15.3)	3	5	1	0	24 (21.8)	14	7	3	0	0.3135
XELIRI + Bevacizumab	–	–	–	–	–	1 (0.9)	1	0	0	0	–
Constipation	42 (71.2)	36	5	1	0	44 (40.0)	43	1	0	0	0.0430
Diarrhea	44 (74.6)	43	1	0	0	51 (46.4)	45	6	0	0	0.3227
Nausea/vomiting	44 (74.6)	44	0	0	0	60 (54.5)	52	7	0	1	0.0124
Stomatitis	41 (69.5)	41	0	0	0	40 (36.4)	37	2	1	0	0.0782
Loss of appetite	43 (72.9)	42	1	0	0	66 (60.0)	52	10	4	0	0.0015
Fatigue	41 (69.5)	34	7	0	0	52 (47.3)	38	13	1	0	0.0010
Rash	36 (61.0)	36	0	0	0	37 (33.6)	37	0	0	0	NA
Fever	37 (62.7)	37	0	0	0	38 (34.5)	37	1	0	0	0.3370
Watering eyes	36 (61.0)	36	0	0	0	32 (29.1)	27	5	0	0	0.0150
Hypertension	57 (96.6)	57	0	0	0	110 (100)	109	1	0	0	0.4794
Peripheral neuropathy	57 (96.6)	49	7	1	0	47 (42.7)	2	39	5	1	1.0040

Abbreviation: XELOX, capecitabine plus oxaliplatin: XELIRI, capecitabine plus irinotecan: NA, not applicable

Primary site of cancer, body surface area (BSA), alanine aminotransferase (ALT) and white blood cell (WBC) were all significantly different between the groups. The grade of HFS was significantly different between the groups. However, there was no difference in each regimen between the groups. The grading scores for constipation, nausea/vomiting, loss of appetite, fatigue and watering eyes were different between the groups (Table 2).

**Fig. 2:**
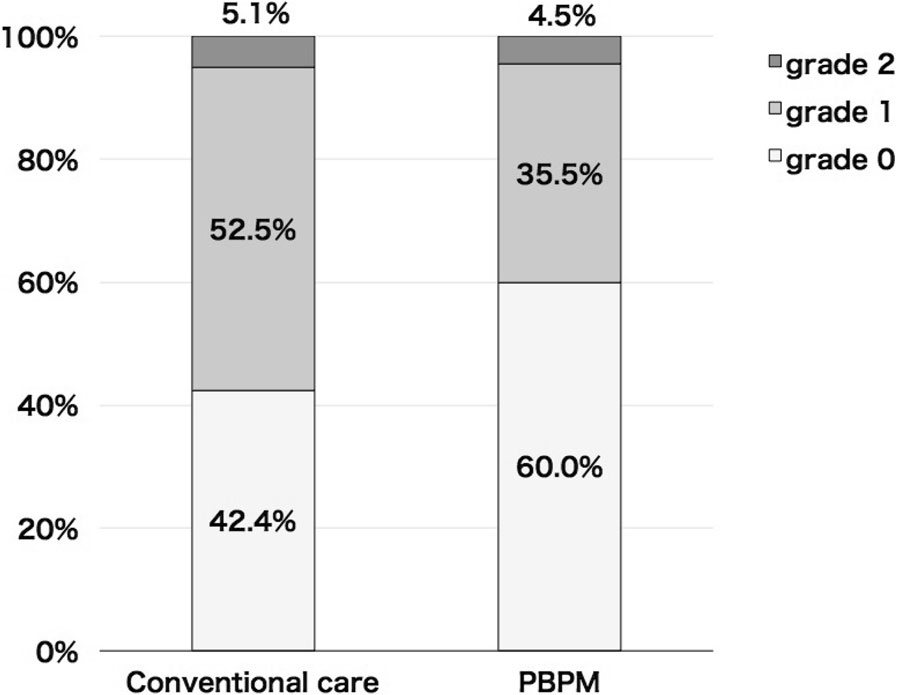
Proportions of HFS grades in conventional care and protocol-based pharmacotherapy management (PBPM)

The proportions of cases of HFS grade 0 in the control and PBPM groups were 42.4 and 60.0%, respectively. The corresponding proportions for cases of HFS grade 1 were 52.5 and 35.5%. The proportions of cases of HFS grades 0 and 1 increased and decreased, respectively in the PBPM group compared to the corresponding proportions in the control group; the intergroup differences were significant (*p* = 0.038) (Fig. 2). HFS grade 2 was confirmed in three of the 59 cases in the control group and five of the 110 cases in the PBPM group. In five cases with HFS grade 2, treatment with steroid creams was initiated based on the PBPM as instructed by pharmacists. Capecitabine treatment as well as treatment of HFS were continued in all cases; the severity of HFS in these cases decreased to grade 1 during the study period.

**Fig. 3:**
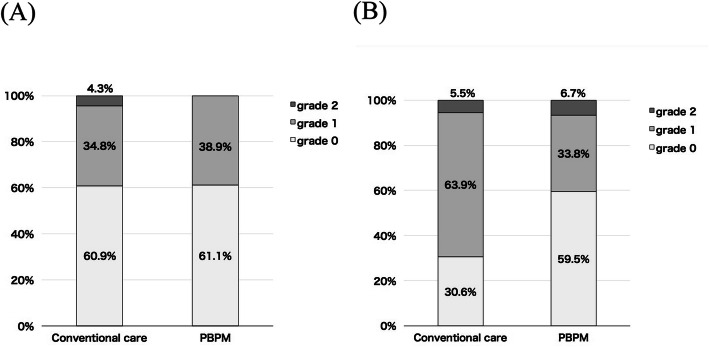
Proportions of HFS grades among patients aged ≤69 years (**a**) and ≥ 70 years (**b**) in conventional care and protocol-based pharmacotherapy management (PBPM)

An ROC curve analysis confirmed an age value of 70 (sensitivity 73.5%, specificity 44.0%, AUC: 0.5700 (95%CI: 0.4175–0.7225)) in conventional care group and 71 (sensitivity 65.9%, specificity 48.5%, AUC: 0.5181 (95%CI: 0.4078–0.6283)) in PBPM group as the cut-off values to prevent an increase in HFS severity. According to the results, we separated the group at the age of 70 years old and the proportions of HFS grades among patients aged ≤69 years and ≥ 70 years in conventional care and PBPM are shown in Fig. 3.

The proportions of HFS grades were similar among patients aged ≤69 years in both the control and PBPM groups, and the intergroup difference was not significant (*p* = 0.891) (Fig. 3a). On the other hand, among patients aged ≥70 years, the proportion of those with HFS grade 0 increased while that of those with HFS grade 1 decreased compared to that in the control group, and the differences were significant (*p* = 0.012) (Fig. 3b).

## Discussion

In this study, we evaluated the effectiveness of PBPM for addressing capecitabine-related HFS in cancer patients by comparing it with conventional pharmaceutical care. In the PBPM group, the severity of HFS was significantly suppressed compared to that in the control group. In addition, PBPM was found to be highly effective among elderly patients (age ≥ 70 years).

Previous research has demonstrated that inadequate medication management among older adults with cancer is associated with poor clinical outcomes, such as increased risk of hospitalization and decreased survival [15, 16]. It has been reported that telephonic follow-up by pharmacists could help achieve higher adherence to maintain the appropriate curative effect with capecitabine [9, 13]. However, in most of the previous reports on pharmacist interventions, capecitabine therapies were focused on monitoring of adverse events through interventions such as a telephonic follow-up. In this study, adverse events were monitored for all patients, and the hospital pharmacist prescribed moisturizing and steroid creams substituting for physicians based on the PBPM. As shown by our results, PBPM was significantly effective and resulted in a 17.6% increase in cases of HFS grade 0 (Fig. 2). In evolving strategies for the management of HFS associated with multitargeted kinase inhibitors including capecitabine, dose reductions or treatment discontinuation until HFS grade 1 or 0 is reached is recommended in patients with HFS grade 2 [17]. However, in all cases of HFS grade 2 in this study, capecitabine was continued, HFS severity improved to grade 1 during the study period, and HFS treatment was not stopped. Therefore, our findings suggested that appropriate instructions by pharmacists could reduce the risk of HFS and HFS severity.

It is of note that PBPM was effective at least in elderly patients (age: ≥70 years); however, no changes were noted in the control group (age: ≤ 69 years). An increased incidence of grade 3 or 4 adverse events was reported in patients aged 80 years or higher receiving capecitabine, whereas the differences in the corresponding incidences were modest in the younger age groups [16]. Because an increased incidence of grade 3 or 4 adverse events was based on moderate renal impairment [16], renal function is considered to be the most important factor affecting the effectiveness of PBPM.

In the PBPM group, the grading scores of nausea/vomiting, loss of appetite, fatigue and watering eyes were significantly increased compared to that in the control group. This result means that especially, early detection and supportive care for these adverse events are required.

There are several limitations of this study. Although we were able to determine the severity of HFS, evidence was unavailable for other adverse events. Furthermore, we did not evaluate progression-free survival and overall survival in this study. Additionally, we were not able to evaluate the renal function in all cases.

## Conclusion

Our findings suggest that PBPM is effective for addressing capecitabine-related HFS among cancer patients aged higher than 70 years so as to avoid an increase in HFS severity.

## Data Availability

All data generated or analyzed during this study are included in this published article.
